# Identification of needs of integrated approaches of occupational health and safety and health promotion

**DOI:** 10.34172/hpp.025.44202

**Published:** 2025-07-15

**Authors:** Yanming Lu, Nektarios Karanikas, Julie-Anne Carroll

**Affiliations:** Health, Safety and Environment, School of Public Health and Social Work, Queensland University of Technology, Victoria Park Road, Kelvin Grove, Queensland 4059, Australia

**Keywords:** Health promotion, Needs assessment, Occupational health, Work

## Abstract

**Background:**

Occupational health and safety (OHS) interventions predominantly target workplace ergonomic, psychosocial, and material risks. Workplace health promotion (WHP) interventions have a primary focus on health education-related activities and health behaviour change. The aim of this study was to assess the workers’ needs of OHS-WHP integrated approaches in Australia.

**Methods:**

A descriptive cross-sectional study was conducted in 2024 among 261 Australian workers across various industries (mainly including education, health, retail, recreation). Eligible participants were employed in Australian workplaces under any contract type and were aged≥18 years. Data were collected through an online survey via Qualtrics platform. This article focuses on the qualitative data generated from the main open-ended question of the survey. The data were analysed employing a thematic inductive approach.

**Results:**

Thematic analysis identified seven key areas for integrating OHS and WHP, mainly including the need for more frequent breaks (49%), enhanced training and education (33%), mental health support (19%), and risk management (19%). The remaining three areas less frequently reported included ergonomic workstations (e.g. adjustable chair) (15%), recreational and physical activities (e.g. stretching exercises) (15%), and Personal Protective Equipment (e.g. visibility clothes) (6%).

**Conclusion:**

Future integrated interventions should prioritise breaks, education, and mental health resources to improve workplace well-being. WHP educational activities could inform OHS information delivery process, possibly enhanced through recreational activities. The engagement of all stakeholders, mainly including employers and workers, when planning and implementing integrated approaches, requires attention and further investigation.

## Introduction

 Worker health, safety, and wellbeing is a very crucial public health topic in Australia and internationally. Globally, around 2.78 million people lose their lives every year because of occupational accidents or work-related diseases, and approximately 374 million nonfatal work-related injuries occur annually.^1^ In Australia, machinery operators and drivers and labourers are at highest risks of work-related fatalities, with workers from agriculture, forestry, and fishing comprising the most vulnerable groups.^2^ Key stakeholders (e.g. employers, policy makers) working in this area, therefore, have been continuously exploring effective strategies to optimise worker health, safety, and wellbeing in varying contexts.^3^ Such strategies can be categorised as occupational health and safety (OHS) and workplace health promotion (WHP) interventions.

 More specifically, OHS interventions predominantly refer to the activities of reducing workplace ergonomic, psychosocial, and material risks (e.g. physical, chemical, biological), thus preventing work-related diseases and injuries.^4,5^ WHP interventions have a primary focus on health education-related activities and health behaviour changes (e.g. lifestyle).^4,5^ An integrated approach combining OHS and WHP elements has been advocated by researchers and practitioners over the past few decades.^6^ The reported benefits of such an approach that involves both individual and work environment aspects encompass effective disease prevention and management, reduction of occupational injuries and disabilities, reduction of healthcare and social costs, improved worker productivity and morale, and long-term intervention gains (e.g. policy and environmental sustainability).^6-9^

 However, to date, within the published literature, there exist scarce systematic research and robust empirical evidence of integrated approaches.^7,9,10^ It remains vastly unclear how to best plan, implement, and evaluate OHS-WHP integrated approaches.^5,7,9^ Despite some emerging evidence reporting on several issues such approaches might address (e.g. nutritional issues, musculoskeletal disorders),^5,7^ it remains unknown whether such issues correspond to what workers actually need. The needs in workers (i.e. intervention recipients) can be referred to as “felt” needs based on the Bradshaw’s typology of four types of needs: felt needs - intervention participants; expert/normative needs - professional bodies; expressed needs - stakeholders who observe the services; and comparative needs - similar attributes).^11^

 For several reasons, it is of primary importance to understand “felt” needs as a starting point when planning integrated approaches. First, practically and theoretically, OHS law and policies refer to the duty of care of employers to staff, including the duty to consult workers, and also emphasise the duty of workers to attend to their own health and safety by complying with reasonable instructions of employers.^12,13^ Yet, it remains unclear whether the actual needs of workers have informed OHS-WHP integrated approaches or those mainly fulfil “expert” views (e.g. OHS professionals). This might analogically reflect public health literature indicating that the community voice appears to be inadequately considered by practitioners when delivering health services, thus contributing to the service ineffectiveness.^14-16^

 Second, beyond the obligations of employers, workers are still expected to play a proactive role in identifying and managing risks in the work environment.^17^ Third, the investigation of the other types of needs (i.e. expert/normative, expressed, comparative) would only be possible if the “felt” needs could be well understood beforehand. Furthermore, when workers report what they need signals a meaningful participatory co-design engagement, which has been extensively employed in implementation science with very good outcomes.^18^

 The above suggest that it is almost imperative to undertake a systematic assessment of worker needs to ensure that their voice is heard and considered by relevant stakeholders, such as employers, OHS professionals, WHP professionals, and government bodies. As such, the workers’ self-reported needs could comprise fundamental evidence to inform how to effectively plan, implement, evaluate, and refine integrated approaches throughout their full intervention cycle. Despite evidence supporting the benefits of integrated OHS-WHP approaches, limited research has explored workers’ self-identified needs. Understanding these needs is critical for designing effective interventions tailored to workplace contexts. Planning and implementing the interventions that rigorously act on the self-reported needs of their recipients could to a large extent improve participation frequency and satisfaction and enhance the effectiveness of interventions.^19^ However, there is a recurring barrier to reporting the actual needs of intervention recipients in the broader context of health interventions.^20,21^ Given the dearth of empirical evidence on integrated OHS-WHP approaches,^10^ understanding “felt” needs in workers, as an important starting point (e.g. particularly when having difficulty finding workable areas for an emerging topic), could help intervention practitioners make informed decisions as to how to prioritise information gathering process and design feasible and meaningful intervention strategies, objectives (short-term goals), and/or aims (long-term goals). As, to date, no published evidence exists regarding self-reported needs of OHS-WHP integrated approaches amongst workers, this study aimed to address this gap by undertaking a national-wide worker needs assessment in Australia.

## Methods

###  Study design 

 This study was part of a broader descriptive cross-sectional study including a tailored survey with a series of open-ended and closed-ended questions. The development of the survey was informed by relevant literature,^9,10^ the PRECEDE-PROCEED model,^22^ published surveys in other contexts,^23,24^ and guidelines about good survey designs.^25,26^ The survey is comprised of four sections, including demographics and questions about workers’ needs of integrated approaches, the extent to which integrated approaches workers receive, and how workers consider the examples of extant integrated approaches identified from a recent scoping literature review.^10^ A specific question at the beginning of the survey asked interested participants whether they consented to participate after reading the respective information about the research.

 To improve the content and face validity of the survey, a pilot study was undertaken in March 2024 amongst ten eligible participants from diverse occupations and demographics to review the structure, content, and length of the survey questions. One key consideration in this process was to assess whether participants could properly understand the definitions of OHS, WHP and integrated approaches. The pilot study participants demonstrated high satisfaction and suggested only minor changes (e.g. choice of sex, the sequence of the questions). The final survey version took approximately 10-15 minutes to complete.

 This study only reports on the data from the central open-ended question listed below, preceded by definitions and examples of OHS and WHP to ensure participants would understand the scope of the two interventions and provide more valid answers. After reading the participant information and consenting to participate, respondents could freely write their answers, click the choice of “unsure”, or skip this question.

####  Introductory text

*From this point on, the survey uses the terms occupational health and safety (OHS) and worksite health promotion (WHP). Although those sometimes can sound similar, please refer to the definitions and examples below to understand what OHS and WHP are about*.

####  Definitions

- *OHS aims to reduce risks by mainly changing the work environments, for example, optimum temperature and noise, control of chemicals, and machine guards*.
- *WHP aims to provide health education and/or promote healthy lifestyles in workers*.
- *Workplace integrated approaches include both OHS and WHP interventions*.

####  Example: worker fatigue

- *OHS: schedule more breaks for all workers in indoor rest areas to manage fatigue*
- *WHP: recreational games to help workers understand how they can individually reduce fatigue at work*

####  Survey question

*Considering the definitions and examples above, can you briefly describe any need(s) or area(s) at your workplace where you believe someone could combine OHS and WHP solutions?*

###  Participants 

 Eligible participants should be currently employed in Australian workplaces under any contract type (i.e. full time, part time, casual), and should be ≥ 18 years of age. Voluntary response sampling was employed for recruitment. Given the descriptive cross-sectional study design, the minimum required representative sample size was 385 Australian workers (margin of error: 5%; confidence level: 95%; population size: 14 115 100 employers in Australia as of September 2023).^27^

 The recruitment strategies consisted of (1) word of mouth (e.g. researchers shared the study and recruitment information through their professional and social channels), (2) online public platforms with incentives (i.e. a draw for five gift cards of AUD $20 each), such as Facebook, Twitter, and institutional website (e.g. ‘Participate in Research’ university webpage), and (3) the Prolific crowdsourcing platform which offered access to research participants who received a predetermined compensation (i.e. AUD $6/each participant for 15 minutes to complete the survey) after they completed the survey and the data were verified by the research team. The Prolific recruitment platform has been considered reliable and useful to recruit participants in academia, with high user satisfaction frequently reported.^28^ For this study, the research team set eligible criteria in the Prolific platform to ensure that the survey respondents had not already participated in this study through the public recruitment campaigns. The Prolific platform then sent email invitations to potentially eligible participants through its internal databases.

 The survey was online and hosted in Qualtrics. Interested participants were able to read the study information and decide whether they consented to participate. Responses to survey questions were voluntary and anonymous. Other than five questions for demographic screening and proper survey flow purposes, no question was mandatory to answer. All qualitative data generated from the survey responses were transferred to Microsoft Excel and Word. Data collection was undertaken in May 2024, with 434 responses initially received. Following data verification, 394 responses were deemed valid. The majority of these valid responses were recruited through the Prolific platform mentioned above, with the recruitment process being quick and smooth. In particular, given that this study provided clear study information via Participant Information Sheet that covered all relevant information, the research team did not receive questions or concerns in the recruitment process, with most participants completing the online surveys in a timely manner and meaningful responses largely received.

 The 394 valid responses were deemed representative to reflect the broader Australian workers; 385 workers were the minimum sample size required for the workforce population of about 14,500,000 employees, 5% margin of error and 95% confidence interval. Regarding the question of interest in this article, there were 261 answers included in the analysis, another 132 respondents clicked the choice of “unsure”, and one did not answer. Table 1 reports the demographics of participants. This lower sample size corresponds to a 6% margin of error, which is slightly higher than the originally set.

**Table 1:** Demographic characteristics of the samples

	**Total survey sample** *** (n*** **=394)** **Mean (SD) or n (%)**	**Sample who replied to the open-ended question and was included in the final analysis** *** (n*** **=261)** **Mean (SD) or n (%)**
Age, mean (SD)	34.42 (11.4)	34.78 (10.9)
Gender		
Male	173 (43.9%)	117 (44.8%)
Female	215 (54.6%)	139 (53.3%)
Other/non-binary	5 (1.3%)	4 (1.5%)
Prefer not to say	1 (0.2%)	1 (0.4%)
Highest level of education		
Never attended school	1 (0.2%)	0 (0%)
Secondary school or senior secondary school	43 (10.9%)	25 (9.6%)
Vocational education and training	39 (10.0%)	23 (8.8%)
Higher education	310 (78.7%)	212 (81.2%)
Missing	1 (0.2%)	1 (0.4%)
Size of organisation		
Large (more than 200 people)	213 (54.1%)	149 (57.1%)
Medium (20-199 people)	96 (24.4%)	63 (24.1%)
Small (0-19 people)	78 (19.8%)	45 (17.2%)
Unsure	6 (1.5%)	4 (1.6%)
Missing	1 (0.2%)	0 (0%)
Is your work mainly office-based?		
Yes	233 (59.1%)	158 (60.5%)
No	161 (40.9%)	103 (39.5%)
Type of industry where participants work		
Education	65 (16.5%)	39 (14.9%)
Health	62 (15.7%)	45 (17.2%)
Retail	52 (13.2%)	27 (10.3%)
Recreation	33 (8.4%)	24 (9.2%)
Government	28 (7.1%)	14 (5.4%)
Professional services	24 (6.0%)	22 (8.5%)
Community services	16 (4.1%)	8 (3.1%)
IT	15 (3.8%)	10 (3.8%)
Finance	14 (3.6%)	9 (3.4%)
Manufacturing	12 (3.0%)	10 (3.8%)
Construction	11 (2.8%)	8 (3.1%)
Agriculture	8 (2.0%)	5 (1.9%)
Technology	8 (2.0%)	8 (3.1%)
Arts	7 (1.8%)	5 (1.9%)
Insurance	6 (1.5%)	5 (1.9%)
Logistics	5 (1.3%)	1 (0.4%)
Energy	4 (1.0%)	3 (1.1%)
Transport	4 (1.0%)	3 (1.1%)
Advertising	3 (0.8%)	1 (0.4%)
Software	3 (0.8%)	3 (1.1%)
Legal services	3 (0.8%)	2 (0.8%)
Science	3 (0.8%)	2 (0.8%)
Aviation	2 (0.5%)	2 (0.8%)
Engineering	2 (0.5%)	1 (0.4%)
Marketing	2 (0.5%)	2 (0.8%)
Mining	2 (0.5%)	2 (0.8%)

*Note:* Missing data mean that participants did not answer the relevant questions.

###  Data analysis 

 All qualitative data were analysed employing a thematic inductive approach informed by the steps described by Braun and Clarke.^29^ More specifically:

1. Familiarising the research team with the data. The research team carefully read and re-read the whole dataset and discussed the nature of the data.
2. Generation of initial codes. The research team assigned the codes to each response, ensuring that the codes closely reflected the original meanings of each response. Of note, a “holistic coding” approach was employed,^30^ that is, assigning not only words and short phrases, but also short sentences to capture the entire meanings of the data. Initial coding was undertaken to gather and collate the answers from participants that best reflected what participants thought an integrated approach might look like. This approach ensured that all pertinent meanings from the data were adequately and rigorously considered and appraised.
3. Generation of themes. The similar or related codes were grouped, leading to the creation of initial themes.
4. Reviewing themes. The research team iteratively discussed, checked, and refined the initial themes prior to finalisation.
5. Defining and naming themes. After the themes were finalised, the research team conducted in-depth analysis to ensure that all themes had clear interpretations.

All researchers were experienced, well-trained qualitative researchers, with their expertise covering OHS, health promotion, and public health. This diverse expertise allowed for the rigorous data interpretation process that closely reflected the original meanings of the responses. Also, the whole dataset was analysed carefully and iteratively to ensure that all responses were adequately considered. One researcher, for example, initially coded the first 50 responses, with the other researchers concurrently coding these responses. All researchers then frequently checked and discussed the codes and data until reaching the consensus. Moreover, where contradictory views occurred, external researchers with relevant expertise were involved to facilitate the discussions until reaching an agreement.

 Thematic analysis was conducted using NVivo software and Microsoft Word, and coding consistency was verified through inter-coder reliability assessments. The research team first independently coded the data and then worked together to compare and discuss the codes. The inter-coder reliability assessment led to a very high consistency (93%), with the disagreements discussed and consensus reached following frequent discussion and revision between the research team and external researchers. This assessment revealed that the responses received were clearly documented, and that the research team conducted rigorous data analysis. This also ensured high dependability in the data analysis process and allowed the findings to be replicated by other researchers. The aforementioned process was undertaken in a repetitive manner in the entire data analysis process, with the themes and interpretations refined accordingly.

## Results

 Seven overarching themes were generated from the 261 responses (Table 2). Supplementary file 1 presents illustrative representative quotations from participants per theme. Overall, the responses varied in length and content richness, ranging from several words to short phrases and paragraphs, and they offered multiple dimensions related to integrated approaches. Some workers reported specific issues and/or areas on which the entire integrated approaches could focus. Other participants clearly portrayed specific intervention strategies for respective OHS and WHP interventions. Some respondents described general intervention strategies for integrated approaches. Only in the minority of responses, however, participants provided contextual information of integrated approaches. As shown in Table 2, thematic analysis identified seven important areas for integrating OHS and WHP, mainly including the need for more frequent breaks (49%), training and education (33%), mental health (19%), and risk management (19%). The remaining three areas less frequently reported included ergonomic workstations (e.g. adjustable chair) (15%), recreational and physical activities (e.g. stretching exercises) (15%), and Personal Protective Equipment (e.g. visibility clothes) (6%). Of note, three areas such as breaks, training and education, and mental health consisted of respective OHS- and WHP-related categories.

**Table 2:** An overview of prevalence of each theme

**Theme**	**N**	**Topics and examples **
**More frequently reported areas**		
Breaks	129 (49%)	OHS-focused breaks (n = 96) e.g. team-based breaks, distance from screens
		WHP-focused breaks (n = 33) e.g. walking, having coffee
Training and Education	86 (33%)	OHS training (n = 51) e.g. toolbox talks, safety practices
		WHP health education (n = 35) e.g. injury prevention, nutrition
Mental health	50 (19%)	OHS approach (n = 27) e.g. job designs, workloads
		WHP approach (n = 23) e.g. counselling sessions, wellness programs
Risk management	51 (19%)	e.g. air conditioning, noise control
**Less frequently reported areas**		
Ergonomic workstations	41 (15%)	e.g. adjustable chair, screen brightness
Recreational and physical activities	38 (15%)	Recreational activities (n = 17) e.g. puzzles, games
		Physical activities (n = 21) e.g. stretching exercises, fitness classes
Personal protective equipment	16 (6%)	e.g. visibility clothes, long pants

###  Theme 1: Breaks

 Of the 261 responses, around half (n = 129; 49.4%) reported on the needs of having breaks in the workplace. These types of breaks could be further classified into OHS-focused breaks and WHP-focused breaks. OHS-focused breaks (n = 96; 36.8%) meant that breaks were required due to work-related factors, or workers explicitly listed them as an OHS topic. In turn, WHP-focused breaks (n = 33; 12.6%) referred to breaks required for non-work-related reasons, or workers clearly marked them as a WHP initiative.

####  OHS-focused breaks

 The majority simply stated that they needed more OHS-focused breaks without providing any contextual information. The reasoning of requiring more OHS-focused breaks included skipping mandatory breaks, high stress, limited opportunities of socialisation, back pain, eye and muscle strain, headaches, overwork, postural issues, fatigue, and unhealthy work environments. The groups needing OHS-focused breaks were often those identified as workers with night shifts, high workloads, extensive standing, challenging situations (e.g. stressful customers, difficult tasks), and limited social interactions.

 Furthermore, some mentioned several forms of OHS-focused breaks, such as information sessions (e.g. courses), team-based breaks, distance from screens, breaks for eating and drinking (e.g. for heat management), naps after lunch, and walking. Others indicated the necessity of having dedicated rooms, such as modern or comfortable break areas exclusively for staff. A few also suggested job-related changes to effectively have a break, for instance, online work meetings via walking with video turned off, hiring more staff to reduce individual workloads, more outdoor work areas, and a walking group.

 Regarding implementation contexts, some preferred to set scheduled breaks in work hours, and some required additional breaks. In particular, some suggested that WHP recreational activities be incorporated into OHS-focused breaks to ensure that mandatory breaks were taken, not skipped, suggesting a potential way WHP could inform OHS.

####  WHP-focused breaks

 Differing from OHS-focused breaks, WHP-focused breaks appeared to be considered an additional, beneficial, and voluntary activity. They could be delivered via various forms, such as walking, games, having coffee, stretching and strengthening activities, meditations, additional breaks to participate in WHP interventions, self-care sessions, and team breaks with no discussions about work. The reported reasoning behind WHP-focused breaks was mainly related to fatigue prevention. Moreover, a few suggested that WHP-focused breaks be implemented with the changes in the work environment (e.g. social activities in a more comfortable break room) and stated the influence of workplace environments and individual behaviour changes. In addition, the minority suggested that occupational health professionals, such as therapists, be involved in supporting personal breaks.

###  Theme 2: Training and education

####  OHS training

 OHS training was a recurring reported area (n = 51; 19.5%), aiming to support worker safety when completing occupational tasks. Different areas and forms of OHS training were stated by the respondents. The activities most frequently mentioned were properly moving equipment and safely completing manual tasks in the workplace, with, however, no detailed contextual information. Some workers mentioned their preference for continual OHS training and information flow, such as frequent and more sessions of OHS training. Preferred ways of delivering OHS training were of particular importance for some participants, such as peer-based communication and appointment-based internal system.

 Of note, some workers mentioned that WHP should have a role in delivering OHS training, such as WHP facilitating the communication of toolbox talks and safety practices, with some key benefits reported to enhance training satisfaction and acceptance. Last, some workers posed that OHS training could be expanded to target not only employees, but also wider community members, such as students at risk and customers more broadly. The above highlight that OHS training may be a joint effort, requiring multilevel participation to enhance worker health and safety.

####  WHP health education 

 Health education (n = 35; 13.4%) was a key focus in WHP activities. Topics related to health education encompassed breaks, personal fatigue management, physical movement (e.g. exercise), proper postures, good mental health, heat management, injury prevention (e.g. slips, trips, falls), living environment, nutrition (e.g. heathy meals), and teamwork. The suggested activities to deliver health education included classes, videos, employee assistance programs, wellness programs, and mindfulness sessions.

###  Theme 3: Mental health 

 Fifty responses (n = 50; 19.2%) were identified regarding improving worker mental health, with two approaches reported including changing the work environment (OHS approach; n = 27; 10.3%) and promoting individual lifestyle changes (WHP approach; n = 23; 8.9%).

 Twenty-seven workers highlighted that mental health should be managed as part of reducing psychosocial risks. Stress was the mental health issue most frequently reported. The minority also referred to the reasons behind poor mental health. For example, one participant reported that more colleagues were warranted to collaboratively deal with difficult situations. However, only a few suggested specific strategies, including setting enforceable boundaries with customers, limiting workloads (e.g. managing challenging cases), and more support from other organisational departments. The above emphasised the necessity of changing the work environment to minimise psychosocial risks.

 Mental health support services in WHP (n = 23) consisted of several forms to facilitate individual lifestyle change, mainly pertaining to counselling sessions, wellness programs, social events, meditations, social opportunities to connect with colleagues, stretching and strengthening activities, and mindfulness sessions.

###  Theme 4: Risk management

 Excluding the psychosocial risks mentioned in the theme above, fifty-one workers (n = 51; 19.5%) identified additional work-related risks, mainly ergonomic and material risks. Temperature control was a recurring topic, mainly concerning the control of air conditioning for ensuring comfortable temperature (e.g. not too cold or hot). Other areas were related to noise control and management, reduction of harmful exposures in health care settings (e.g. animals, surgical equipment, interactions with patients, chemicals, radiation), risk assessments for working from home, injury prevention (e.g. slips, trips, falls), and administrative controls to limit meeting duration. Furthermore, some reported on strategies of improving the facilities in the workplace to minimise material risks, including, for example, improving the quality of office environments, having access to gym and healthy food facilities, and meeting in a shaded area to avoid sun.

 Interestingly, some listed risk assessment (normally considered an OHS process) as a WHP activity, potentially suggesting an avenue of integration. One response, for example, indicated that the management of excessive noise generated by workers (e.g. conversing loudly between them or over the phone) should be addressed in WHP (e.g. constant reminders), and such issue was always ignored by the company. This may provide an important question about how to design and deliver the effective messaging to ensure that relevant stakeholders could properly assess the risks, receive the correct information, and maintain safe practice at multiple levels.

###  Theme 5: Ergonomic workstations

 Forty-one (n = 41; 15.7%) reported on the needs of utilising ergonomic workstations. Half of them described specific equipment, including adjustable desks, monitors, chairs, and adjustment of screen brightness. The reasons behind varied, consisting of high workload, headaches, discomfort when sitting, extensive sitting, postural issues, eye and neck strain, and back pain. Of note, approximately half of the 41 respondents reported only the short phrase “ergonomic workstations” or similar without stating any specific equipment.

 Moreover, some workers pointed out the necessity of ensuring proper ergonomic setup in varied work contexts, for example, a home visit to check ergonomic workstations for employees working from home. Some highlighted that ergonomic setup should suit individual needs, such as workers whose workstation designs should also account for the needs of their customers. Medical practitioners, for example, may need special workstations (e.g. booster chairs) when treating children. Interestingly, a few believed that ergonomic workstations, typically classified as OHS interventions, could be a sole health promotion effort, potentially suggesting that WHP may have a unique role in offering ergonomic design and setup.

###  Theme 6: Recreational and physical activities

 Exclusively classified as WHP, recreational activities (n = 17; 6.5%) included, for example, puzzles, coffee walks, games, and lunch with colleagues. Reported benefits of recreational activities comprised increased social connections between workers, stress and fatigue reduction (e.g. eye strain), active participation in adequate breaks, and injury prevention.

 Besides, physical activities (n = 21; 8%) included physical moving during work hours (e.g. short physical activity routine), fitness and exercise classes, games about movement, and stretching and strengthening activities. The reasoning behind physical activities mainly included extensive sitting, back pain, postural issues, and disconnection with colleagues.

###  Theme 7: Personal protective equipment 

 Solely referring to as an OHS area, sixteen responses (n = 16; 6.1%) pointed out the necessity of utilising personal protective equipment (PPE). Some simply stated the phrase of “PPE” or similar without listing any specific equipment. Nonetheless, others mentioned specific items for enhancing their safety, including, for example, high visibility clothes, long pants and shirts, hard toe shoes, heat proof gloves, bottles, umbrellas, safety gear (e.g. trolleys), noise-free headphones, hand wash products, and sun cream. Of note, a few listed that noise-free headphones may be also considered a WHP activity, suggesting a potential way of integration.

 Although PPE was the theme less frequently reported compared with other themes, it might reveal some improvement areas, particularly when performing physical and outdoor work. These might include, for example, limited availability and utilisation of PPE, individual and environmental challenges to perform PPE (e.g. knowledge gaps, behavioural challenges, peer influence), and other support services required to supplement PPE.

## Discussion

###  Overall picture

 The findings from this study highlight seven overarching themes: breaks, training and education, mental health, risk management, ergonomic workstations, recreational and physical activities, and PPE. These areas adequately reflect what Australian workers actually need from integrated OHS-WHP approaches, particularly for the industries of health, education, retail, recreation, and professional services and large size of organisations that were more frequently represented in our sample.

 Nonetheless, one third of the 394 survey respondents declared ‘unsure’ when answering the question of interest in this study. Although we did not collect further data about this, there might be some implications. For example, these respondents may not have sufficient knowledge to express their needs, or may lack in motivation to answer the question, a similar finding supported by Waters et al.^31^ Nonetheless, the fact that two third of the participants offered their perspectives suggests that currently most Australian workers appear to demonstrate health and safety literacy skills.

 The above would serve as a very promising starting point, given that the success of any type of interventions would not be possible if recipients have a poor understanding of intervention products.^32^ By extension, it suggests that the majority of Australian workplaces may show positive attitudes towards integrated approaches. Given that OHS management typically involves enforceable actions to control risks,^13^ such OHS interventions could be considered as entry points for intervention practitioners to engage with potential workplaces to implement integrated approaches. For instance, to receive the initial support of integrated approaches from workplaces, intervention practitioners could start to evaluate existing OHS interventions to identify their weaknesses and then design WHP to address them.

 On the other hand, several workers from this study mentioned the mechanisms of how OHS-WHP integrated approaches could work theoretically, that is, the work environments and individual behaviour changes influencing and informing each other. Not only could this finding inform the planning process of integrated approaches, but it could also be used to refine OHS and WHP curricula. Currently, although modern health promotion programs are considered multidisciplinary, and aim to target multiple determinants of health, a strong emphasis ties in health service delivery and individual behaviour change for various reasons (e.g. limited budget, lack of expertise, political reasons).^33^ Therefore, health promotion curricula, for instance, could be more advanced if including more technical skills related to the changes of physical environments, such as practice in timely hazard identification, effective risk assessment and implementation of occupational hygiene measures. In turn, OHS could involve more content related to intrapersonal psychological change at the individual level to enhance worker health and safety.

 Nevertheless, to successfully implement integrated approaches, employer support is considered a very important facilitator.^9^ One interesting observation from this study is that the roles of employers (e.g. leadership involvement) were not mentioned across almost all responses. This may possibly suggest that there might exist limited appreciation in Australian workplaces of the influence of employers when implementing integrated approaches, notwithstanding that OHS regards more enforceable and legislative activities, whereas WHP is mainly a voluntary activity and requires more considerations.

 Rojatz et al,^34^ for example, highlighted the facilitators of a successful WHP implementation, including the economic status of the work environment, employer support, quality of intervention strategies, implementation process, participants’ mental perceptions, and evaluation methods. Nonetheless, it is worthwhile noting that the involvement of employers during the delivery process might cause negative impacts. For instance, Junker et al found that employer involvement in deploying WHP interventions might cause privacy concerns and discomfort amongst employees, thus contributing to less engagement and low satisfaction.^35^ Future research should explore how workers perceive the roles of employers in the intervention planning and implementation processes, and investigate the employers’ views about OHS-WHP integrated approaches.

###  Examples of integration opportunities

 The findings of this research, indeed, highlight several integration opportunities. For instance, the findings reveal that ergonomic workstations, although typically a common OHS risk control in office environments, are still warranted in integrated approaches. Extensive evidence suggests that ergonomic workstations are beneficial to reduce the negative consequences of musculoskeletal disorders, physical inactivity, and overwork.^36^ In our dataset, the above extended to employees working remotely (e.g. home), with respondents suggesting this occupational group requires careful consideration (e.g. home visits for ergonomic setups). Similarly, individual needs in a hybrid work environment should be considered in the intervention planning and implementation processes. These findings are also supported by Davis et al^37^ and Dugar et al.^38^

 Besides, one interesting area identified from this study was that some workers listed ergonomic setup as a WHP activity. This provides the potential avenues that WHP could assist in the educational activities related to correctly utilising ergonomic workstations. Further, Lee et al^39^ observed that ergonomic workstations should be combined with lifestyle changes to more effectively reduce the risks of musculoskeletal disorders. This is because ergonomic workstations may not correct all postural issues.^39^ A complementary effort, WHP, could enhance the overall worker health and contribute to longer-term health and safety impacts. Such combination may be first based on a quality evaluation of ergonomic workstations in order to identify suitable lifestyle goals.^40^ For example, where neck pain could not be entirely reduced by ergonomic workstations, regular neck exercises should be encouraged.

 As another example of integration opportunity, half of the participants from this study focussed on breaks. Interestingly, the types of breaks varied in terms of number, reasoning, form, and workplace. Although evidence suggests that having a break is normally considered an individual behavioural activity,^41^ our research reveals that simply encouraging workers to have more breaks as an individually-initiated activity may not be an effective approach. Instead, an integrated approach to support adequate breaks should target both environmental and individual changes.

 The findings above raise important considerations for Australian workplaces. First, there might exist non-individual and less modifiable barriers to taking adequate breaks. It is important that workplaces provide more support to understand the causes of inadequate breaks and take reasonable steps to act on these causes at the organisational level. Second, the Fair Work Ombudsman sets specific rules regarding work hours and number of breaks for employees in various industries.^42^ Still, our study findings indicate that inadequate breaks could be very common in Australian workplaces. This could be attributed to the fact that breaks are usually considered relatively low-risk, self-motivated health and safety activities.^43^ Also, workplaces (e.g. employers, health and safety professionals) may view “break” differently in terms of subjective importance and relevance,^44^ and may regard it as a personal issue rather than a central safety and health effort.

 There exists little evidence-based research into the workplace factors associated with inadequate breaks and the extent of modification of these factors.^45^ Also, there is a paucity of evaluation and monitoring frameworks in Australia for addressing relatively low-risk health and safety issues.^45,46^ In particular, Australian industries that are perceived as low OHS risk profile appear to ignore the necessity of transparently reporting strategies to improve worker health and safety,^46^ and these low-risk issues may be escalated further.^47^ As it was outside the scope of this study to examine why workers required more breaks, future research should investigate more systematically why staff perceive breaks as inadequate, existing barriers to having adequate breaks, and the level of modification of such barriers. This will provide a direction that needs urgent intervention efforts.

###  WHP informing OHS training 

 From this study, some OHS training activities were marked by some workers as WHP. This suggests that (1) there is a potential preferred approach at the participant level to incorporate WHP into OHS training, or (2) workers associate training with WHP more than with OHS. This can be explained by the fact that WHP has a primary focus on health education, disease prevention, and lifestyle change.^4,5^ Still, it is unlikely that WHP can completely or extensively replace OHS training. Instead, WHP might inform OHS training in terms of innovative delivery modalities and communications.

 Similarly, growing evidence suggests that traditional, formalised information sessions are likely to lead to low engagement with and limited participation in health services.^32,48^ Innovative health education activities should be engaging, interesting, and easily accessible to generate high satisfaction.^49,50^ The findings from this study suggest that innovative health education approaches with recreational activities could be incorporated into OHS training, risk management (e.g. behavioural factors), and mental health promotion. The goal would be to design and deliver more effective messaging to support safe and healthy practices at the individual and organisational levels (e.g. the application of intrapersonal, interpersonal, and ecological models^22^ under WHP for scale-up integrated approaches). This provides another interesting area for further intervention research.

## Policy Implications

 Policymakers should consider mandating structured break schedules and incorporating mental health support within workplace safety guidelines to address the identified needs. In Australia, the relevant policy support in addressing these two areas is related to the recent code of practice of managing psychosocial hazards.^51^ This affords fundamental guidelines to increase intervention efforts in the real world to focus holistically on workplace health and wellbeing, rather than heavily on safety aspects. Yet, there exist significant gaps. First, current guidance emphasises the importance of effective communication and management processes, but it does not visibly suggest clear and specific health-related roles and responsibilities for relevant stakeholders (e.g. health care and health promotion professionals) particularly in improving worker health and wellbeing, rendering WHP absent and challenging. This warrants an urgent policy effort to formally acknowledge and encourage joint contributions between clinical medicine, public health, and OHS.^52^

 Second, given that workplace health and wellbeing is influenced by work-related and non-work-related factors with interactive relationships,^53^ policymakers should carefully consider and mediate the roles, responsibilities, and interests of all relevant stakeholders, with extensive stakeholder consultations, effective and timely policy development and revision, and ongoing monitoring systems warranted in future. One key consideration is that policies should be comprehensive and ethical, allowing careful and thorough considerations of all scenarios (e.g. workplace health accidents, perceived mental health needs, balanced approaches between tool-based assessments and individual health/personal experiences).

## Study Strengths And Limitations

 To the best of our knowledge, this study is the first national-wide research to systematically understand workers’ needs of integrated approaches in Australia with the employment of a relatively representative sample. Although the number of the responses that answered the questions was relatively lower than initially targeted, thematic saturation was achieved. Moreover, although some of the grey literature has highlighted several areas integrated approaches might address,^54,55^ the respective definitions and scoping of OHS and WHP can be inconsistent, meaning that such findings may not reflect the workers’ real needs of integrated approaches. This study addressed this gap, first providing clear definitions of integrated approaches and then collecting data about worker needs.

 Further, there exists very little empirical evidence on needs assessments amongst intervention recipients in the specific context of integrated approaches. This study through an anonymous survey offered workers a chance to freely express their needs, promoting public engagement in service design and delivery. Moreover, the findings from this study are not only beneficial to workers, but also useful for policy makers, employers, legislative stakeholders as they are based on first-hand data and contribute to the evidence base for integration initiatives. Finally, given that the distinction of respective OHS and WHP interventions is clear from a definition viewpoint, this study provides directions of how WHP transcends discipline boundaries of OHS. Still, our dataset did not offer in-depth and detailed contextual information of these needs. Future research should explore the above through in-depth discussions (e.g. focus groups and interviews).

## Conclusion

 This study highlights important areas that need to be considered in the planning process of integrated approaches, including breaks, training and education, mental health, risk management, ergonomic workstations, recreational and physical activities, and PPE. In Australia, having adequate breaks amongst workers could be the primary focus in integrated approaches. Not only should breaks be considered an individual behavioural prevention effort, but there may also exist less modifiable barriers to taking breaks at the organisational level. Future legislative and research efforts should focus on relatively low-risk health and safety issues.

 Integration opportunities were mainly identified for WHP educational activities informing OHS information delivery process (e.g. proper utilisation of ergonomic workstations, risk communication, OHS training). Health education approaches with recreational activities, as part of WHP, could also show promise in enhancing the delivery process of OHS training. Importantly, although the engagement of employers when planning integrated approaches may be another useful entry point, their involvement in the implementation process requires further investigation. Last, apart from the “felt” needs in workers we analysed in this research, future studies should consider expert/normative needs of professional bodies and expressed needs of all stakeholders as fundamental and meaningful factors during the planning process of any OHS-WHP integration intervention.

 Employers should prioritise structured break policies, integrate health education into OHS training, and create supportive environments for mental health to foster a healthier workforce. Future management efforts should focus on supporting integrated approaches at multiple levels. For policymakers, future policy development should consider more consistently health aspects of employees, rendering employee health-related guidelines clear, comprehensive, feasible, reasonable, and ethical. For health practitioners, future intervention efforts should enhance the inputs of preventive health and health promotion in the workplace setting, with meaningful collaborations required with safety professionals, employees, employers, and policymakers.

## Competing Interests

 The authors declared no conflicts of interest.

## Data Availability Statement

 Data supporting this research are available from the corresponding author upon reasonable request.

## Ethical Approval

 The study was approved by Queensland University of Technology Human Research Ethics Committee (Ethics Number: 8350).

## Supplementary files

Supplementary file 1. Themes and illustrative representative quotations from participants
