# Supplementary material for: Identification of needs of integrated approaches of occupational health and safety and health promotion
Source: Health Promot Perspect. 2025 Jul 15;15(2):153–64. doi: 10.34172/hpp.025.44202 (PMC12450578; doi:10.34172/hpp.025.44202)
Supplement: Supplementary file 1 — Themes and illustrative representative quotations from participants [file hpp-15-153-s001.pdf]

Lu et al, **Health Promotion Perspectives.** 2025;15(2):153-S1
doi: 10.34172/hpp.025.44202
https://hpp.tbzmed.ac.ir

Supplementary file 1. Themes and illustrative representative quotations from participants

Theme	N	Topic	Illustrative representative quotes
Breaks	129	OHS-focused breaks (n = 96)	• Break to reduce stress, comfortable rest area to reduce stress (P1)<br>• Recreational games to ensure OHS compulsory breaks implemented not skipped (P11)<br>• More breaks from the computer screen and adjust screen brightness (P34)<br>• Relaxing and resting area for quick nap after lunch (P50)<br>• Reduce sitting and screen time: online meetings via walking with video turned off/mobile phones (P120)
		WHP-focused breaks (n = 33)	• Burn out because of specific patients such as dementia, or an aggressive patient that is causing physical harm to nurses (P3)<br>• Extra 10-min break to reduce fatigue (P18)<br>• Recreational games during these breaks (P30)<br>• Provide information sessions with specialists that detail ways you can reduce fatigue and discomfort (P193)<br>• More break to participate in WHP (P246)
Training and Education	86	OHS training (n = 51)	• Equipment to assist in lifting heavy parcels & educate how to use & store equipment (P21)<br>• Cleaners need to put away equipment (P25)<br>• Manual handling injuries, appropriate height reaching ladders (P31)<br>• Proactively talk with workers to see if they are ok with the call (P33)<br>• An appointment-based system with internal access only, with students with risks (P42)<br>• Not lifting heavy items, seek assistance if required (P94)<br>• Increased safety education sessions (P95)<br>• Constant education of safety management (P99)<br>• Train OHS, training for employees (P111)

Theme	N	Topic	Illustrative representative quotes
			• Manual handling (P119)<br>• Training: move large groceries (P121)<br>• Trolleys to move large equipment, and online and in-person demonstrations to correctly use the equipment (P128)<br>• Supplements to boost the immunity to prevent the spread in customers (P143)<br>• Customers need to carry items properly from the warehouse near the workplace (P169)<br>• Correct lifting techniques (P185)<br>• WHP embedded into existing OHS Toolbox Talks (P228)<br>• More interesting toolbox talks about safety (P237)<br>• Education of safety practices at home in WHP (P243)
		WHP health education (n = 35)	• Importance of looking after health (P54)<br>• Slips, trips and falls: education of prevention (P66)<br>• Educational videos on fatigue importance (P76)<br>• Lifestyle education (P96)<br>• Diet, drinking, regular exercise-related information (P98)<br>• Education: regular stretching & moving around (P110)<br>• WHP: more awareness of surrounding environment (P179)<br>• Exercise class, education (P251)
Mental health	50	OHS approach (n = 27)	• No social interaction for medical practitioners (P12)<br>• Stress management because of high pressure patients or high workloads to reduce mistakes (P48)<br>• Stress (P68)<br>• Worker stress: hire more staff (P114)<br>• Mental health: more than one staff member to deal with difficult situations (P148)<br>• Safe and supportive work environment (P177)<br>• Workplace stress (P254)
		WHP approach (n = 23)	• Stress management workshop, counselling & wellness program (P1)<br>• Social events to reduce stress (P114)

Theme	N	Topic	Illustrative representative quotes
			• Mental health resources, stress management workshops, access to counselling services (P177)
Risk management	51		• Clear job role (P1)<br>• Noise management (P69)<br>• Risk management: from animals, surgical equipment, interaction with owners & chemicals, x-rays/imaging (P73)<br>• Temperature management (e.g. better heating and cooling) (P101)<br>• Clutter/boxes in the pharmacy to reduce tripping hazards (P108)<br>• Noise reduction from nearby construction (P125)<br>• Clear work division in workers (P137)<br>• Noise when doing medication rounds, paper charts (P152)<br>• Temperature control: comfortable office temperature (P197)
Ergonomic workstations	41		• Provide the right equipment for good sitting posture (P4)<br>• Adjusting workstation & ergonomic equipment to reduce the risk of musculoskeletal injuries, however, home visit to check setup is preferred for working from home (P6)<br>• Ergonomic chair, adjustable monitor and desk (avoid long sitting, comfort when typing, eye level to reduce neck strain) (P51)<br>• Ergonomic chair (P192)<br>• Medical practitioners, for example, may need special workstations (e.g. booster chairs) when treating children (P219)
Recreational and physical activities	38	Recreational activities (n = 17)	• Fitness class, coffee walk & mini game (P1)<br>• Scheduled game time (P5)<br>• Recreational games to reduce fatigue (P43)<br>• Puzzle (P45)<br>• Posture, bending & stretching (P81)<br>• Fun staff lunch (P104)<br>• Games in recreation room with refreshments (P212)
		Physical activities (n = 21)	• Physical exercises (P24)<br>• Take a walk (P72)

Theme	N	Topic	Illustrative representative quotes
			• Encourage more moving to reduce long sitting (P103)<br>• Badmintons (P133)<br>• Physical activity & fitness: virtual fitness classes, step challenges (P138)<br>• Back pain from sitting to much (P153)<br>• Times for a walk because of sitting too much (P164)<br>• Physical activity: on-site fitness facilities, group exercise (P197)
Personal protective equipment	16		• Better quality hand wash liquids (P32)<br>• Hard toe shoes (P37)<br>• Wear well fitted footwear to reduce any injuries and damage (P66)<br>• Sun cream (P67)<br>• High visibility clothes (P70)<br>• PPE safety gear (P128)<br>• Bottle/umbrella for self-care in different weathers (P222)
